# Freak to queer bodies: affirming the grotesque in contemporary art

**DOI:** 10.3389/fsoc.2025.1435207

**Published:** 2025-07-24

**Authors:** Júlia Mello

**Affiliations:** Laboratory of Landscape Studies, Federal University of Espírito Santo, Vitória, Brazil

**Keywords:** contemporary art, grotesque, body, gender, queer, freak shows, Fernanda Magalhães, Laura Aguilar

## Abstract

This article analyzes artworks that employ the grotesque to challenge hegemonic discourses. It draws a parallel between bodies depicted in 19th-century freak shows and queer bodies represented in artistic productions from the mid-1990s to the early 2000s. In both historical and modern contexts, bodily difference plays a pivotal role in defining normality. Various artistic endeavors have aimed to reconsider this premise, particularly by addressing gender issues, exemplified in the works of Brazilian artist Fernanda Magalhães and Chicana artist Laura Aguilar. This investigation employs image analysis, focusing on art history and visual culture, alongside a literature review. The findings suggest the potential for rethinking the social status of bodies labeled abnormal and highlight the political impact of contemporary art in promoting social inclusion.

## 1 Introduction

“The freak is thus neither unusually gifted nor unusually disadvantaged. He or she is not an object of simple admiration and pity but is a being who is considered simultaneously and compulsively fascinating and repulsive, enticing and sickening.” (Grosz, 1996, p. 56)

“When people with stareable bodies […] enter into the public eye, when they no longer hide themselves or allow themselves to be hidden, the visual landscape enlarges.” (Garland-Thomson, 2009, p. 9)

“Thinking queer means to question, problematize, and challenge all well-behaved forms of knowledge and identity. Queer epistemology is, in this sense, perverse, subversive, impertinent, irreverent, profane, and disrespectful.” (Silva, 2010, p. 107, *translated by the author*).

The fat body, along with many others deemed strange or abject, is often categorized as a grotesque form, encompassing deformities, monstrosities, and unresolved states. This classification places such bodies within the discourse of abnormality, extensively explored by Foucault, notably in *Abnormal: Lectures at the Collège de France 1974–1975* (2001). Intolerance, curiosity, and prejudice are just a few of the reactions triggered by these bodies, which persist in their peculiarity and their inherently grotesque mode of existence.

Reflecting on these bodies prompts us to inquire into how they transcend the boundaries of normality and why they evoke discomfort. How do these representations negotiate visibility and marginalization? In what ways does the grotesque function as a means of resistance and empowerment in queer artistic practices? To explore these questions and more comprehensively, we must delve into the realms of history, cultural studies, and the “anthropology of deformities” (Courtine and Vigarello, 2011), while also examining social deviations. The objective of this investigation is to further elucidate the queer connection with Fernanda Magalhães and Laura Aguilar, and to guide their utilization of the grotesque as a provocative strategy.

Queerness has an anti-normative character and embraces anti-aesthetics, offering new positions to those who have been excluded and silenced by dominant culture. It is within this context that many of the living bodies inhabiting this planet have resisted being subalternized by the capitalist, petro-sexual, and racial regime (Preciado, 2023). Articulating the fat body’s rebellion through the grotesque allows for a reconsideration of queerness’s potential in generating new perspectives on corpulence. Thus, the guiding hypothesis of this research is that queerness can be understood through the genealogy of freaks. These bodies, once categorized as aberrations and marked by physical difference, were excluded from everyday life and revealed to the public only in spectacles. Now, they move through the public sphere with empowerment, embracing their strangeness. To illustrate these theoretical perspectives, we can briefly introduce Fernanda Magalhães and Laura Aguilar, whose practices vividly embody the intersection of the grotesque and the queer.

Fernanda Magalhães is a Brazilian artist who utilizes corpulence to confront normative standards, disrupting conventional boundaries. She frequently exposes her naked body in photographs, videos, and performances, provoking the audience with her nudity in public. Her body size, perceived as “strange” and “anomalous” through clinical eyes, challenges societal norms and values. She often collaborates with other artists, especially individuals who are fat, transgender, and Black, in performances that emphasize collective action and strategies of subjectivity.

Magalhães identifies as a lesbian and incorporates cuir^1^ elements into her artistic endeavors. Her engagement with the grotesque involves using her body to challenge dominant cultural and beauty standards discourses regarding corporality. Magalhães’ self-referentiality deliberately departs from the idealized portrayal of the female body prevalent in traditional art history. This portrayal, characterized by art historian Clark (1956) as “restrained” and “balanced,” epitomizes aesthetic beauty and stands as a quintessential symbol of Western cultural norms.

By advocating for autonomy in self-representation, the artist prompts us to contemplate Smith and Watson’s (2012) insights, thereby introducing novel avenues for understanding the bodies of (fat) women. Through her artworks, Magalhães employs grotesque forms that induce ambiguity, thereby obfuscating the delineations between peripheries and focal points. This deliberate blurring of boundaries enables a deeper exploration of their inherently ambivalent character.

The artist began developing her artworks during her travels to Rio de Janeiro in the 1990s, where she used photography to document the oppression she faced as a fat woman in a city that upheld ideals of bodily “fitness.” Complementing her photographic work, she later incorporated elements of collage and cutouts, consistently intertwining her self-image with the societal construction of female bodies perpetuated by media and visual culture. Notably, Magalhães integrates performance as a significant component of her artistic endeavors, characterized by a series of actions involving installations, dancing, and public interventions, often executed collaboratively.

These proposals bear striking resemblance to those of the American artist Laura Aguilar, who identified as Latina due to her Mexican heritage and who magnifies in her photographs the struggles for Chicana^2^ and lesbian causes. A fundamental feature of her poetic endeavor is the emphasis on ethnic-racial issues, primarily informed by queer theories. Aguilar’s photographs are notable for unveiling her nude, corpulent body across diverse landscapes, frequently alongside other marginalized bodies, suggesting an integration with natural surroundings.

Just as in the case of Magalhães, Aguilar offers a critique of body standards within the context of art history, employing excesses to subvert the construction of the idealized, “sealed,” “contained,” and “purified” female nude. The *Bakhtinian grotesque*^3^ (1987) manifests in the artist’s photographs, characterized by degradation, which entails the valorization of corporeal and material elements, merging with the earth, the lowly. There is a pronounced advocacy for the amalgamation of the body with natural landscapes, with carnality, with excesses, and with all that is grandiose. Aguilar’s corporeal representation is visceral, borrowing from the term utilized by art historian Jones (2000), and provocative; it navigates modesty while evoking a sense of peril. It embodies the grotesque, following the framework of Harpham (1976, 2006): a complex interplay of attraction and repulsion.

Magalhães and Aguilar artworks help propose a correlation between freaks and queer, positing that the display of bodies deemed anomalous, prevalent from the mid-19th century to 1940 in Europe and the United States (Bogdan, 1996), intersects with queer misfit bodies, illustrating that bodily difference plays a crucial role in shaping the concept of normalcy. By considering those aspects, this investigation intertwines the concepts of “freak” and “queer” through an analysis of images within the contexts of art history and visual culture, supplemented by a literature review. The study includes an examination of the documentary *Rotundus* (2005), which features artworks by Magalhães, as well as *Gorda 8* (1995) and Laura Aguilar’s photography *Grounded #111* (2006).

## 2 Methods

This study employs a qualitative research approach, integrating methodologies from art history, visual culture, and queer theory to analyze the intersection of the grotesque and non-normative bodies in contemporary art. The research is structured around three primary methods: image analysis, literature review, and critical discourse analysis.

### 2.1 Image analysis

A key component of this study is the examination of visual materials, particularly the artworks of Fernanda Magalhães and Laura Aguilar. The analysis focuses on how these artists employ grotesque aesthetics to challenge hegemonic discourses on body normativity. The study considers elements such as composition, framing, subject positioning, and intertextual references within their works. Furthermore, this research draws from the theories of Bakhtin (1987) on the grotesque body and Jones (2012) on self-representation to contextualize the artists’ visual strategies. The study includes an examination of the documentary *Rotundus* (2005), which features artworks by Magalhães, as well as *Gorda 8* (1995) and Laura Aguilar’s photography *Grounded #111* (2006).

### 2.2 Literature review

This research is supported by a comprehensive literature review, drawing from key texts in art history, visual culture, queer theory, and body politics. Foundational works by Foucault (2001) on normativity and biopolitics, and Garland-Thomson (1996) on freak studies provide a theoretical basis for the study. The review also integrates insights from Fiedler (1996) on the cultural construction of freakery and Goffman (1988). Additionally, the study incorporates discussions on fat activism and body politics, drawing from Cooper (2016) and Snider (2018). The grotesque as a theoretical framework is examined through the works of authors such as Bakhtin (1987) and Harpham (1976, 2006), while Grosz (1996) provides crucial insights into bodily ambiguity and feminist corporeality. Preciado’s (2010) perspectives on queer theory and embodiment further support the study’s analysis.

### 2.3 Critical discourse analysis

To examine the broader cultural and sociopolitical implications of grotesque representations, the study employs critical discourse analysis. This method allows for an exploration of how dominant narratives construct and regulate bodily difference. It also considers how Magalhães and Aguilar’s works subvert these narratives by reclaiming agency through visual representation. Drawing on the “anthropology of deformity” (Courtine and Vigarello, 2011), this study also considers reflections on how medical discourse, spectacle, and normativity intersect in the historical construction of deviant bodies. The notion of normativity is understood as a system that regulates bodies and identities (Foucault, 2001), shaping their visibility and legitimacy. The analysis extends to media portrayals, exhibition contexts, and academic discourse to assess the impact of these artworks within contemporary discussions on queerness and the grotesque.

## 3 Queer and freak contexts

Sexual and identity normativity is intrinsically linked to the regulation of bodies within a social system that privileges certain forms of existence while marginalizing others. As Foucault (2001) argues, modern societies operate through disciplinary and biopolitical mechanisms that define and maintain corporeal and identity norms. Goffman (1988) expands on this discussion by analyzing how deviations from the norm are socially stigmatized, creating symbolic hierarchies between acceptable and unacceptable bodies. In this sense, the hegemony of normative identities is not sustained solely through explicit coercion but also through cultural and discursive mechanisms that reinforce the naturalization of certain gender and sexual standards. Preciado (2010) and Butler (2003) demonstrate how the performativity of gender^4^ and sexuality can serve as a site of contestation, destabilizing fixed and normative meanings imposed on dissident bodies. By articulating these perspectives, it becomes possible to understand how normativity not only excludes but also regulates modes of existence, rendering queer and grotesque bodies subject to selective processes of visibility and marginalization.

Building on this framework, the concept of queer emerges as a critical lens through which to challenge and deconstruct these normative structures. Queer can be translated, in the words of Cooper (2016), as simultaneously referring to a sexual preference and to a quality or sensibility of dislocation within society. Queer is characterized by its antinormative nature and its appreciation for anti-aesthetics, offering novel stances for individuals excluded and silenced by dominant cultural norms. Queer theory emerges in the late 20th century from a nexus of political, economic, and social relations, seeking to reframe the position of marginalized groups in contemporary Western society.

Jagose (1996) introduces queer theory, highlighting its wide-ranging connotations. Depending on context, it can denote dissonance, carry homophobic undertones, or serve as colloquialism. In recent discourse, queer has been extensively applied to embrace sexual minorities and underpin a theory emerging from gay and lesbian studies. Jagose emphasizes queer as a fluid and evolving concept rather than a fixed construct. Its ambiguity and adaptability, as noted by scholars like Edwards and Graulund (2013), position it as a significant aspect of the grotesque. What intrigues scholars about queer is its defiance of normative discourses and its use of elements considered anti-aesthetic to challenge the prevailing cultural framework.

Art historian Amelia Jones conceptualizes queer as a fluid and destabilizing category that challenges identity essentialisms and social norms. The author seeks to connect queer theory with performance and contemporary art, arguing that the body and its representations are central to the construction of subjectivity and queer politics (Jones, 1998, 2000, 2002, 2010, 2012, 2016, 2017, 2020; Jones and Campbell, 2020). For Jones, queer operates as a form of resistance to normativity, disrupting fixed structures of identity, particularly those based on gender and sexuality. Drawing on thinkers such as Butler, the author emphasizes the performativity of gender and sexuality as mechanisms for unsettling established norms.

Another key aspect of Amelia Jones’ approach is the performativity of the body in art. Jones examines how performance art functions as a space of resistance and subversion, highlighting artists such as Ron Athey (1961–) and ORLAN (1947–), whose works challenge the boundaries of gender, flesh, and identity (Jones and Campbell, 2020; Jones, 1998). Moreover, the author’s research contests traditional notions of objectivity and authorship in art, arguing that queer theory destabilizes the idea of fixed meaning, reinforcing the significance of the spectator’s subjectivity in the interpretive process. Jones also explores the relationship between queer theory and the materiality of the body, emphasizing that queer theory extends beyond sexual identity to encompass various forms of non-normative embodiment. This study follows this perspective, considering how fat, racialized, and modified bodies challenge normative categorizations and broaden the understanding of body politics in contemporary art.

Similar to “queer,” “freak” is a term conventionally perceived as offensive, comparable to “faggot” or “nigger.” Literary critic Fiedler (1996) observes that there is significant prejudice surrounding the freak domain, to the extent that mixing its elements with “high” culture is not accepted. Gerber (1996) suggests that “freak” encompasses a range of characteristics, including deformities, disfigurements, disabilities, and excesses. Bogdan (1996) proposes a more comprehensive delineation, indicating that definitions consistently originate from a physical perspective and encompass various conditions such as dwarfs, giants, hairy individuals, extremely thin people, amputees, microcephalics, fat individuals, albinos, conjoined twins, individuals with supernumerary organs, hermaphrodites, individuals with dermatological conditions, tattoos, and any anatomical variations.

The individuals engaged in freak shows delineated a more nuanced classification: “born freaks” (those naturally born with anomalies), “made freaks” (individuals who acquired freak status, such as tattooed or fatness), and “novelty acts” (persons showcasing abilities like sword swallowing or contortionism). Additionally, there existed a category of false freaks, termed “gaffed freaks,” comprising individuals feigning monstrosities through illusionist techniques and makeup (Bogdan, 1996). Philosopher Grosz (1996) posits that the definition of freaks is as intricate as that of queer. She perceives the term as a political act, recognizing it, akin to queer, as a word that can be employed for both confrontational purposes and self-assertion.

Freak pertains to the domain of the grotesque, inducing both horror and fascination simultaneously, exhibiting ambiguity and challenging binary oppositions.^5^ As indicated by Grosz (1996), it represents the amalgamation of the human with the animal (“The Camel Woman,” “Julia Pastrana, the Monkey Woman,” “Jo-Jo, the Dog Boy,” “The Elephant Man”), the human with another human (“The Tocci Brothers,” “Chang and Eng”), nature with culture (“Wild man of Borneo”), one gender with another (bearded women, intersex people, Joseph-Josephines, Victor-Victorias), adults with children (dwarfs), humans with deities (giants), and deceased with living (skeleton man).

Garland-Thomson (1996) traces the historical trajectory of the term “freak” in modern discourse, noting its evolution from signifying “wonder” to connoting “error.” Initially, “freak” emerged to describe sudden or unusual changes in plants, as evidenced by John Milton’s usage in the poem “Lycidas” (1638), where it referred to a spot of color. Over the course of the 17th century, its meaning expanded to encompass notions of “whimsy” or “fancy.” The *Oxford English Dictionary* (1989) records the phrase “freak of nature” (referring to an abnormally developed individual) as of 1847, indicating its adoption to denote any form of anomaly (Garland-Thomson, 1996). Within the same historical context, Bogdan (1996) notes the emergence of the term “freak show” to describe exhibitions featuring individuals with physical differences, commonly staged in circuses, fairs, carnivals, and other entertainment venues.

Against this backdrop of historical and cultural context, the artworks of Magalhães and Aguilar emerge as powerful interventions that challenge prevailing norms and narratives surrounding the body. Through their respective practices, these artists disrupt conventional understandings of corporeality, offering alternative perspectives that celebrate difference and defy categorization. Magalhães, with her bold explorations of corpulence and identity, confronts societal norms with unapologetic defiance, while Aguilar’s intimate and introspective photographs invite viewers to reevaluate their perceptions of beauty and belonging.

A closer examination of Magalhães and Aguilar’s artworks reveals their transformative potential in reshaping our understanding of the body and its place within society. By reclaiming agency and visibility for marginalized bodies, these artists challenge us to embrace diversity and inclusivity in all its forms, forging new pathways towards a more equitable and compassionate world.

### 3.1 Affirming the grotesque in contemporary art (part I)

The documentary *Rotundus*^6^ (2005) appears to draw upon certain aspects of the concept of the freak by incorporating works by Magalhães that reference the photographic styles of Diane Arbus (1923–1971) and Joel-Peter Witkin (1939–). Arbus’s photographs, produced during the 1950s and 1960s, depicted individuals with physical deviations, eliciting a perceptual shock that highlighted the otherness of the deformed body. Her oeuvre captured various subcultures, including the worlds of carnivals,^7^ wax museums, middle-class families, as well as themes of death and illness, resulting in what can be described as “anti-portraits”^8^ (Santos, 2006).

As will be elucidated in the ensuing discussion, fat ladies held significant prominence within the context of freak shows, notably from the latter part of the nineteenth century onwards. As per the insights of Bosworth (1984), a biographer of Diane Arbus, the artist demonstrated a profound and nearly voyeuristic fascination with freak shows. Acting as an observer, Arbus purportedly engaged in extensive photography, capturing images of a fat lady whom she had meticulously observed for hours during a visit to Hubert’s Freak Museum. This establishment, situated between Broadway and 42nd Street, boasted a longstanding history of over 25 years (Bosworth, 1984).

In addition to Arbus, Joel-Peter Witkin is one of the references used by Magalhães. Witkin’s bodies are excessive, mutilated, ambiguous, fragmented, folded, and manipulated. They exhibit a dark peculiarity, set in disturbing and provocative scenes. The fat body is a recurring element, as evidenced in works such as *Sanitarium, NM* (1983), *Metropolis, NM* (1981), *Portrait of the Holocaust* (1982), and *Woman as the Measure of All Things* (1982, Figure 1).

**Figure 1:**
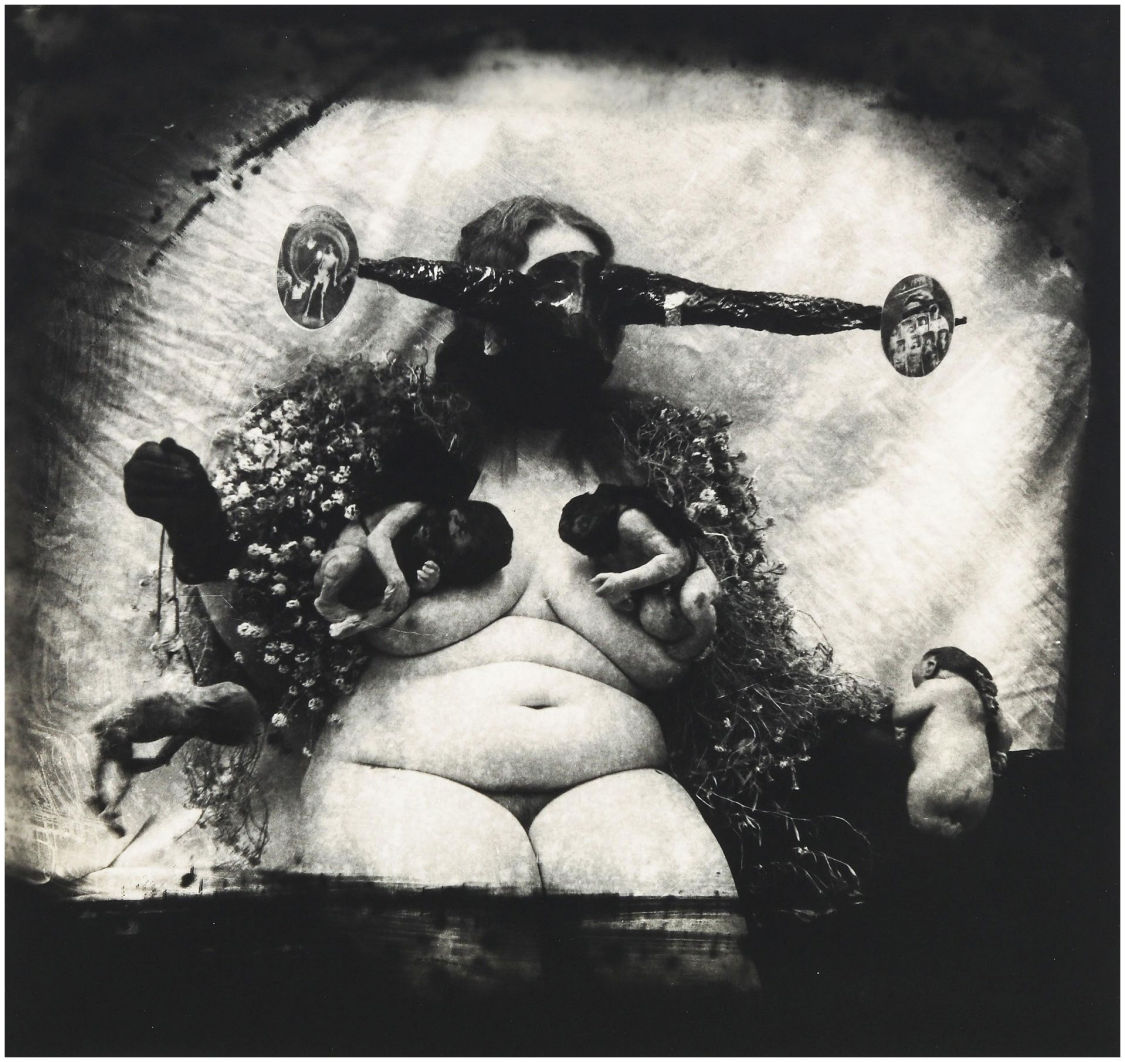
Joel-Peter Witkin, *Woman as the Measure of All Things*, 1982. Photography. Etherton Gallery, Tucson. Available at: http://www.artnet.com/artists/joel-peter-witkin/woman-as-the-measure-of-allthings-a-5VQTh31S51Me8f6EaeZpwA2. Last accessed on: May 18, 2024. Copyright Joel-Peter Witkin, courtesy of Etherton Gallery.

*Woman as the Measure of All Things* is a disquieting artwork, and each viewing reveals a new element. The title suggests a reference to the phrase by the philosopher Protagoras (490 BCE–420 BCE), “man is the measure of all things,” but with “man” replaced by “woman.” In this possible interpretation of Protagoras, Witkin presents a woman, a kind of goddess, holding a fetus in one hand. On the other side, there is a baby with congenital malformations. The central figure is covered in ornaments: a cloak of flowers interspersed with veiled babies, suspended over her breasts. The woman’s gaze, from behind a bizarre mask composed of cutouts from other images, is haunting. A more detailed analysis reveals a skeletal, unbalanced figure on the left side of the mask and a thin women in coffins on the right side, reinforcing the dark, macabre, and gloomy nature of the work.

**Figure 2:**
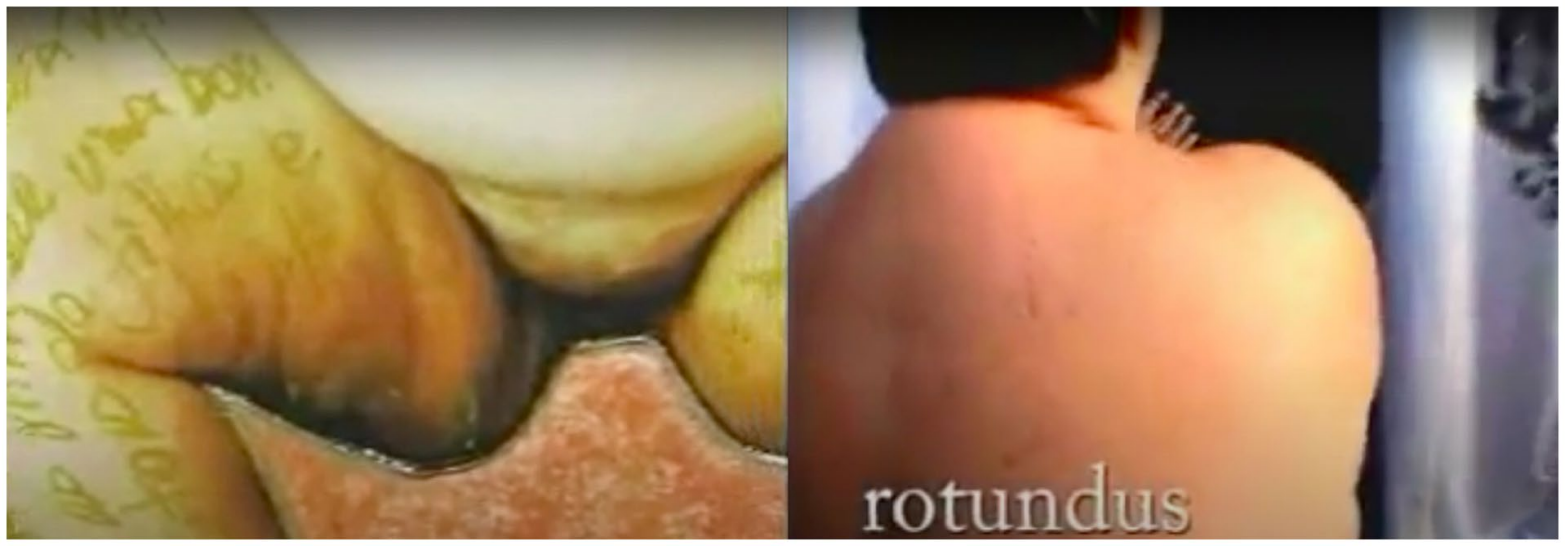
Fernanda Magalhães, *Rotundus*’ frames, 2005. Available at: https://www.youtube.com/watch?v=0DDknRfJBZU. Last accessed on: May 18, 2024.

*Rotundus* incorporates these disturbing elements through the aesthetic and artistic narratives and experiences of Magalhães. This non-fiction short film was produced during the Kinoarte Documentary Workshop in Londrina and won the Udihara Trophy for Best Screenplay at the Londrina Festival (2005). The body depicted in the documentary, much like those portrayed by Arbus and Witkin, is characterized by framings and perspectives that evoke the grotesque. As a cinematic work, *Rotundus* serves as a potent social discourse, functioning as both an act of resistance and a form of critique. By exploring unconventional aesthetics, Magalhães challenges conservative notions of bodily beauty (Figure 2).

As depicted in the frames, the scenes emphasize corporeality and the portrayal of fatness, interwoven with Magalhães’ narratives. These elements suggest a continuation of the *contaminated photographs*,^9^ a hallmark of the artist’s creative process stemming from the series *The Representation of the Nude Fat Woman in Photography* (1995). *Rotundus* juxtaposes images, sounds, and noises, interspersed with voice. The “contamination” of audiovisual effects serves as a political device that reinforces Magalhães’ protest. Moreover, connections can be discerned with cinema and Dadaist photomontages, particularly considering films such as Hans Richter’s *Filmstudie*^10^ (1926) and the collages of Hannah Höch (1889–1978), a German artist involved in the Dada movement.

Magalhães’ artworks, more than her spoken words, leave a profound impact on the documentary, offering fresh perspectives and new avenues of exploration. Unlike static images, her moving works focus on specific body parts and textual fragments, which, when magnified, underscore the political dimensions of the “freak” body. While scholars like Lockard (2012) and Kuusinen (2006) may reject the notion of associating the fat body with the grotesque, viewing the works of Arbus and Witkin as explorations of cultural diversity, the documentary illustrates the potential of employing the *politics of ugliness*^11^ (Rodrigues and Przybylo, 2018) as an active means of addressing the invisible, the unspoken, and the immeasurable. This approach involves conceiving of “ugliness” as a politically charged terrain engaged in dialogue with various forms of difference, including gender, ableism, race, class, age, sexuality, health, and body size.

**Figure 3:**
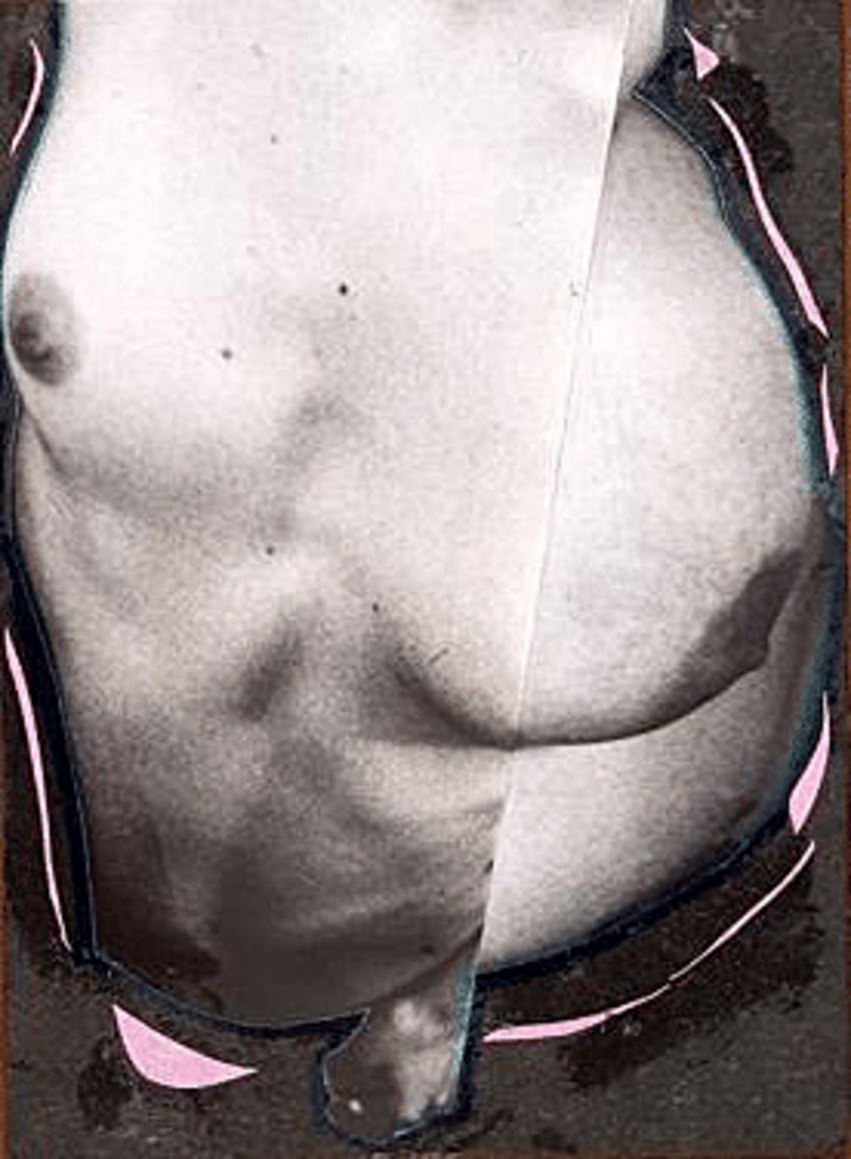
Fernanda Magalhães, *Gorda 8*—*The Representation of the Nude Fat Woman in Photography*, 1995. Mixed technique. Available at: https://www.flickr.com/photos/fernandamagalhaes/3020016044/. Last accessed on: May 18, 2024.

The most impactful pieces in the documentary are those from the series *The Representation of the Nude Fat Woman in Photography*. Intertwined with one another, set to a haunting soundtrack, and focusing on erogenous zones, they demonstrate that fatness encompasses much more than just appearances. The peculiarity of the bodies resulting from the photomontages, and later from the animation enhanced with various audiovisual techniques, prompts us to recognize these practices as strategies for positioning “unconvetional” bodies to challenge contemporary modes of perception (Berger, 1999). These perceptions are often influenced by a visibility economy that favors the male sex and gender. In this context, the emphasis on *Gorda 8* (Figure 3) is not coincidental.

Two bodies vie for the same space, one engulfing the other; they merge into one another. The black and white photography dulls the gaze, imparting a sense of coldness. The contrast between the slim and the overweight torsos becomes stark. Ambivalence, uncertainty, strangeness, and unease overwhelm us. In the video, the artwork briefly appears, moving vertically, right after Magalhães’ statement: “it’s a difference that people are bothered by.” The artwork itself enables us to perceive dissent coincidentally through the lens of agonistic struggle, as per Mouffe’s framework (2015). In her view, the agonistic model acknowledges the impossibility of eradicating conflict. When applied to interpreting the image, this suggests a rupture in the symbolic representation of the “normal” female body. Hence, we can infer that there’s a manner of expressing oneself “agonistically,” revealing alternatives to prevailing bodily norms.

*Gorda 8* is inherently queer: it’s transgressive, challenging, provocative, and freakish. The image captures the disjointed overlaps, glue smudges, pink paper outlining the photographs, and a hand blurring the boundaries between the two bodies. The result—a hastily assembled, “careless” collage—evokes a protest akin to the works of Hannah Höch, as we previously suggested. One possible interpretation of Magalhães’ ambiguous work is the transition (or communion?) from the slim body to the fat one. Regardless, these are incomplete bodies, “open,” as Bakhtin (1987) might suggest—an interplay between self and other, as Freud (1996) would posit. They are abject because they are ambiguous, neither bodies nor objects, as Kristeva (1982) describes, and formless because they offer constant exchange between contrasting parts, akin to Bataille’s project (1929). All these grotesque facets touch upon the freak universe and can also be contemplated in works like *Aus der Sammlung: Aus einem ethnographischen Museum (1929) by Hannah Höch*^12^.

Similarly to *Gorda 8*, this piece is part of a series consisting of 17 photomontages created between 1924 and 1930. In this image, we observe a body structured by a tribal mask, a baby’s torso, and an eye purportedly from a woman, sourced from a magazine. The hybrid creature, seemingly on display, serves as the artist’s retort to the politics and culture of the Weimar Republic, which marginalized women, ethnic/racial minorities, and children. According to researcher Antle (2007), Hannah Höch was undoubtedly attuned to the exclusionary mechanisms of her era, consistently seeking resistance against prevailing ideologies. Many of her works address the theme of ethnographic museums, challenging stereotypical views, particularly regarding race and gender, and contesting the fetishization of exhibiting bodies deemed exotic.

### 3.2 From freak to queer: a sociocultural overview

If we delve into history, we’ll notice that the fascination with bodies deemed unusual is incredibly ancient. This phenomenon is evident within the realm of the grotesque, where we encounter enduring icons like cyclopes, giants, and dwarfs. Records of monstrous births can be traced back to cave drawings and even to the “Šumma izbu” (an Assyrian compendium of omens). Rare and exceptional bodies served as tools for entertainment and profit, even in pre-capitalist societies like the Egyptian, Roman, and European monarchies, which often kept dwarfs and jesters as pets. They also played central roles in the popular festivals of the Middle Ages and the Renaissance, as discussed by Bakhtin (1987). Their exhibition in fairs and markets was commonplace, and in England, under certain circumstances, bodily difference could even be punishable by death if there was suspicion that it resulted from a relationship between a human and an animal (Garland-Thomson, 1996).

Garland-Thomson argues that the discourse surrounding the freak is deeply intertwined with and reflective of the cultural transformation of modernity. Similarly to Courtine and Vigarello (2011), she highlights a significant shift: the once wondrous monster becomes pathologized. There’s a noticeable change in perspective, moving away from celebrating enthusiasm and amusement towards eliciting pity and rejection. Garland-Thomson (1996) emphasizes the fluidity of the relationship between the fantastical and what we now perceive as anomalous and teratological.

The scientific perspective relies on the notion of objectivity, emphasizing regularity over exceptionality and prioritizing empiricism over the fantastical narratives prevalent in the centuries leading up to modernity. The emergence of freak culture stems from rituals that stylize, silence, differentiate, and distance individuals whose bodies are commodified and exploited by both “freak hunters” and showmen. Paradoxically, this process simultaneously categorizes all anomalies into an amorphous realm of alterity (Garland-Thomson, 1996). As Gerber (1996) notes, the freak show can be viewed as both a commercially-driven social entertainment phenomenon and a product of unequal social relations, oppression, and exploitation.

The freak body evokes a sense of disquiet; it is simultaneously strange and familiar, known yet unknown, reminiscent of Freud’s concept of the *unheimlich* (1996). It demands explanations, inspires representations, and provokes societal regulation, serving as a benchmark for what is considered human. Despite the myriad transformations in discourse surrounding anomalous bodies throughout history, the persistent need to articulate, confine, and rationalize these unforeseen corporeal manifestations remains steadfast. While Garland-Thomson (1996) posits that “similar to female and slave bodies, the monstrous body exists in society to be exploited for someone else’s purpose” (Garland-Thomson, 1996, p. 2), Bogdan (1988, 1996) views the exhibitions of freaks as opportunities for empowerment, removing them from the victimhood narrative.

Several critical points are essential for understanding the transformation of the freak phenomenon from “monstrosity” to “illness.” This examination will substantially contribute to our comprehension of the profound stigmatization of corpulence throughout the 20th century. Courtine and Vigarello (2011) posits that the apex of showcasing abnormalities occurred in the 1880s. These exhibitions, referred to as “exotic diversions” or “morbid pleasures,” were integral components of the public display mechanisms for differences, anomalies, deformities, illnesses, mutilations, and human body monstrosities. These spectacles marked the earliest manifestations of the modern entertainment industry, laying the groundwork for the complex interplay between societal norms and the representation of physical diversity.

The 19th century was characterized by a quest to uncover the underlying essence of monstrosity lurking behind minor anomalies, deviations, and irregularities. Courtine emphasizes that these events lie just over a century in the past, appearing distant—a bygone era of popular amusement characterized by an archaic and often cruel exercise of curious observation. According to the author, these sensibilities no longer resonate with our own. Until the early decades of the 20th century, monstrosity was integrated into familiar forms of entertainment, gaining popularity through exhibitions showcasing individuals perceived as living phenomena.

As science assumed control over these bodies, they were also exhibited in glass jars—classified as teratological specimens. Additionally, there were the “exotic morphologies,” the primitive rituals of “human zoos,” and indigenous villages, all of which reinforced the discriminatory perception of bodies, turning racial differences into spectacles—anomalous monstrosity masked beneath an aura of exotic strangeness (Courtine and Vigarello, 2011). This occurred within the context of colonial expansion, which perpetuated the erroneous notion of a “natural” hierarchy of races, wherein the indigenous, primitive, and exotic were contrasted with the “civilized.”

In the 19th century, the figure of the monster encapsulated collective anxieties. Alongside its portrayal in wax museums, at fairs, and even preserved in glass jars, it was appropriated by the anthropology of peril as an exemplar for delineating the physical and moral attributes of criminals. Moreover, it found its place in theatrical productions, often depicting gruesome crimes. This pervasive presence served as an educational tool, illustrating what constituted the norm by embodying its antithesis. Foucault (2001) argues that at the turn of the century, the formation of normalization power relied on exhibition devices that showcased the “wrong” to underscore and valorize the “right.”

According to Courtine and Vigarello (2011), freak shows in France were nomadic and precarious, never integrating with the grand traveling circuses or the established curiosity museums in the capitals. In England, however, freaks were exhibited in amusement parks, with one of the earliest major curiosity museums founded there in 1812. In 1840, in New York, Phineas Barnum (1810–1891) initiated the trade of freaks within an entertainment hub that featured various performances including dance and theater. The Barnum Museum marked the dawn of a new era in spectacles, functioning as the Disneyland of teratology (Courtine and Vigarello, 2011). By the mid-19th century, morphological oddities were subjected to standardized presentation methods, primarily for entertainment purposes.

In the European context, especially in England and France, the exhibition of freaks began to be banned around 1880. Courtine and Vigarello (2011) notes that by 1883, the Elephant Man could no longer appear in public. Hospitals became the new stage for anomalous bodies, and beliefs that monstrosity was a divine or diabolical manifestation, a grotesque product of female imagination, or a result of human-animal relations gradually faded away. Medicine, biology, genetics, and embryology took over, discovering that mutations could be induced in laboratories or result from chemicals, radiation, and industrial pollution.

Courtine argues that during the 19th century, a new sense of compassion emerged, shaping the contemporary view of bodily deformities. The recognition of the human nature of monsters by teratology contributed to this shift, and the legal sphere was evidently affected. Monsters were no longer to remain “outside the law.” The changing perception of the monstrous body led to the understanding that deformity was not comedic but entailed suffering and shame. As Foucault (2001) presents, the monster was seen as a disruption of natural law, impacting and shaking civil, canonical, and religious rights.

The freak gradually distanced itself from popular attractions due to police censorship or compassion. The monstrous body began to be seen as diseased, receiving orthopedic treatments, and being integrated into the “more conventional” labor market. Additionally, it is important to remember that eugenics developed from the late 19th century until 1940, propagating the fear of degeneration. Francis Galton (1822–1911) founded this program, which aimed to enhance the racial qualities of future generations by eliminating the undesirable and multiplying the desirable. The French physician Richet (1919) argued that in human societies, the most intelligent, strong, and brave should have privilege over those who were weak, effeminate, and foolish (“mols, efféminés et bêtes”), lamenting the favoring of the mediocre, sick, poor, miserable, and diseased (Richet, 1919, p. 29).

**Figure 4:**
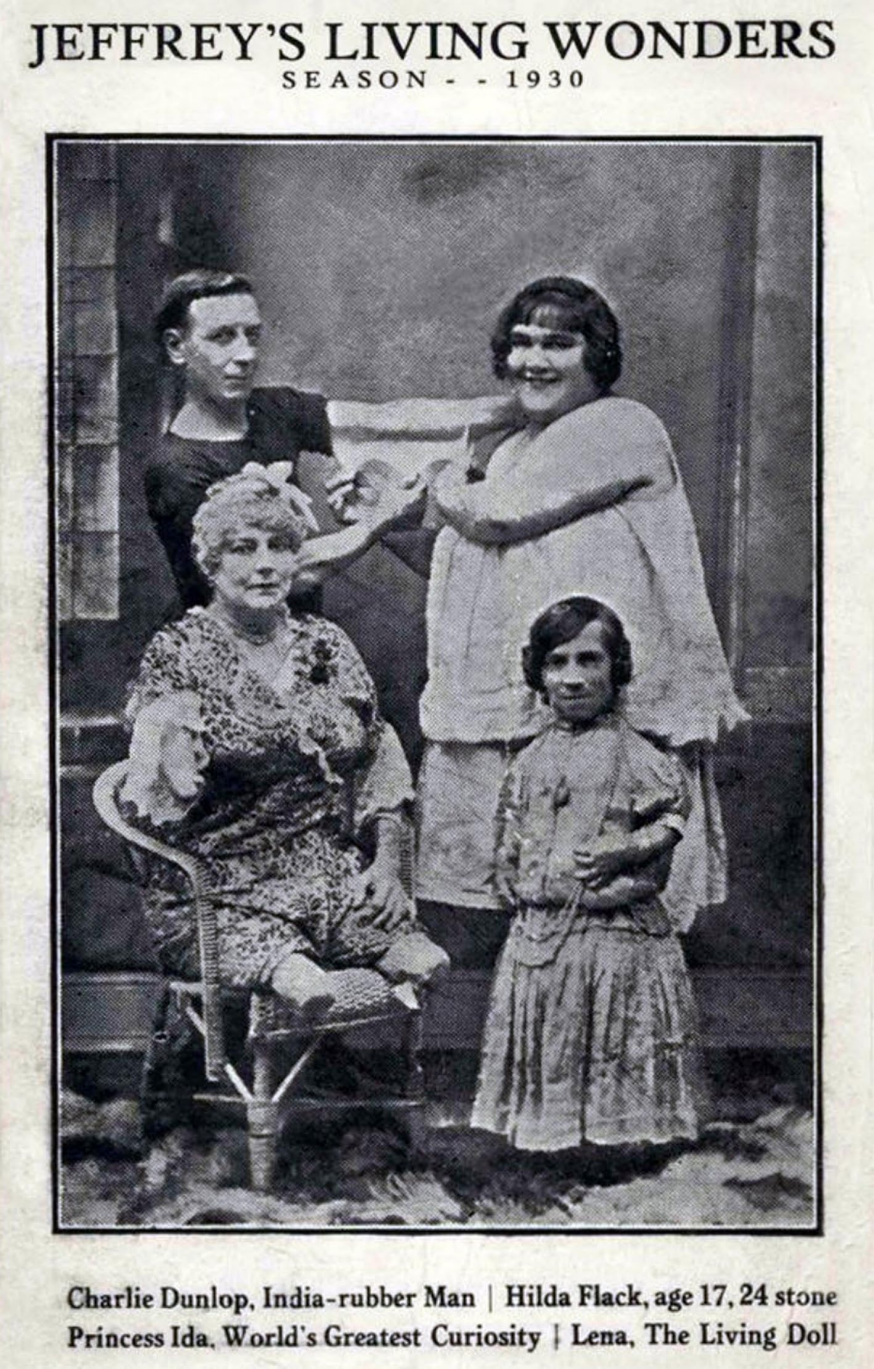
Promotional business card from Jeffrey’s Living Wonders, from the 1930 season. Back: Charlie Dunlop (Indian Rubber Man), Hilda Flack (fat lady). Front: Princess Ida and Lena (Living Doll). Available at: sideshowworld.com. Last accessed on: May 18, 2024.

Nevertheless, from the late 19th century onward, especially in North America, fat ladies gained popularity, and freak shows typically included at least one act featuring them (Figure 4).

**Figure 5:**
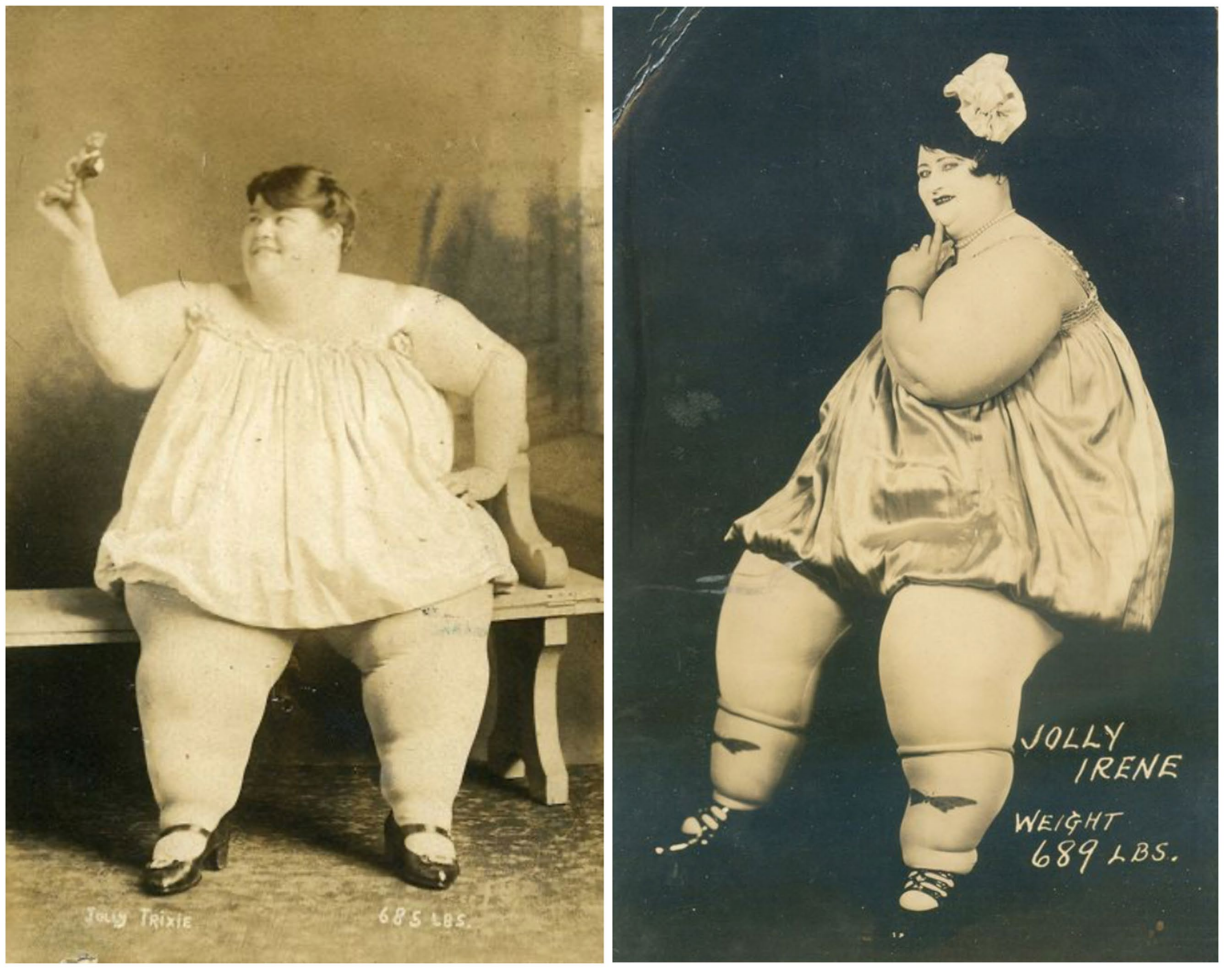
Unknown authorship, Jolly Trixie, [190-] (left) and Jolly Irene, 1920 (right). The author’s collection, 2020.

A brief examination of the presentation of freaks in fairs and shows is warranted. Despite their bodies constituting a defiance of natural laws, the setting often soothed the gaze, with elaborate costumes and tranquil, made-up faces. By the 1860s, specialized studios emerged for capturing the bizarre, and postcards and calling cards served as keepsakes of the fleeting encounters with these phenomena in the parks. Competition among the fat ladies was intense, leading to practices such as misrepresenting their weight, wearing padded clothing, or using posters with exaggerated drawings to draw more attention from the audience, as noted by Haslam and Haslam (2009). According to Gerber (1996), many performers showcased their fatness by wearing revealing attire while seated on small chairs for audience observation. Postcards from that era provided a preview of what spectators could expect to see (Figure 5—left and right panels).

The juxtaposition of the flesh’s magnificence with the small, delicate hands, and the feet constrained in shoes defied the laws of physics as they bore the entire weight. There appeared to be a consistent pattern in the presentation: meticulously styled hair, voluminous elastic dresses, stockings, and a dramatization of (presumed) femininity in gestures. Numerous fat ladies seized the opportunity to shift the notion of mockery away from their bodies, employing the grotesque as a form of provocation, as exemplified by figures like Dainty Dotty (1909–1952) and Alice Wade (1894–1955). Their stage personas often featured nicknames with sexual connotations and incorporated scenes of nudity, elements that became integral to their performances. In some instances, they even permitted audience members to touch them, sparking reconsiderations regarding the societal perception of the fat female body.

Another prominent figure in the realm of fat ladies was Katy Dierlam (1950–2006), a contemporary performer in freak shows. According to Braziel and Lebesco (2001), Dierlam entered the eccentric world of these shows with a deeply political sensibility. Embracing the character of Helen Melon, she used her role as an actress and performer to confront social issues surrounding her body, ultimately gaining a sense of self-assurance. For Dierlam, accepting a performance as a fat lady meant shedding any sense of hiding, embracing her full self without reservation. What might have been considered an “anomaly or deficiency” in everyday life could be transformed into an act of resistance or protest within the realm of these shows (Braziel and Lebesco, 2001).

### 3.3 Affirming the grotesque in contemporary art (part II)

A comparative examination of the photographs featuring fat ladies alongside *Grounded #111* by Laura Aguilar^13^ reveals the significant shifts in the display and visibility of corpulent women in contemporary settings. Additionally, when viewed through the lens of visual culture, which seeks to interconnect images from various domains, we can expand our understanding of art history by recognizing that “[…] the processes of producing meaning are inherently social” (Knauss, 2006, p. 100, translated by the author).

Despite the chronological gap between them, the images served different purposes. While photographers captured the fat ladies for promotional postcards, Aguilar chose self-representation within the realm of art. The performers adhered to a predetermined set of presentations typical of freak shows, as suggested by Bogdan (1988). In contrast, Aguilar positions herself unclothed amidst natural scenery, highlighting autonomy over her corporeality. As Nochlin (1999) and Smith and Watson (2012) suggest, contemporary modes of self-representation offer new avenues, enabling artists to engage in the process of social and cultural construction while navigating their identities as artists, women - and, we can add, as Latina and lesbian, as Aguilar does.

According to Smith and Watson, female artistic expressions throughout the 20th century were shaped by cultural policies of self-representation, paving the way for new perceptions of female bodies. Building on these possibilities, Aguilar presents a series of photographs where she appears to blend with objects from natural settings, notably emphasizing rocks. In *Grounded #111*, her imposing figure is juxtaposed with a large stone in Joshua Tree National Park. Unlike the smiling, joyous depiction of fat ladies in postcards, Aguilar is portrayed with her back turned, her body and form blending into and contrasting with the rocky landscape. The angle of her head suggests introspection and solitude, diverging from the cheerful demeanor of the postcard images.

Jones (2012) suggests that photographic self-portraits serve as a “visual autobiography,” challenging the voyeuristic structures shaped by the male gaze on female bodies. This intrusive mode of observation, reinforcing the hierarchical dominance of privileged subjects, is confronted by Aguilar. This confrontation is particularly evident when examining another photograph by the artist, *Nude, n. 7* (1996), as noted by Jones: Aguilar invites the male gaze while simultaneously resisting its penetrating effects by pressing her body against the earth. In Aguilar’s narrative, she assumes roles as both subject and object, disrupting conventional modes of body observation and their associated implications. Rooted in the scene, she blends in and stands out simultaneously, presenting her back to the audience while asserting her presence, thus generating a conflict in perception modes, and showcasing the defiance of forms.

The ambiguity arising from interpreting representations of fat ladies can be approached through an analysis of Aguilar, despite potential deviations in observation. In these images, there’s a peculiar blend of the strange and the familiar, invoking an uncanny sensation. While viewers may recognize the “everyday” poses and delicate gestures in the ladies’ images, their bodies, cataloged simultaneously as both spectacle and medical anomaly, evoke discomfort, fueling curiosity for entertainment. A contemporary perspective transforms these postcards into unsettling artifacts. Courtine and Vigarello (2011) reminds us that compassion and fear of “monsters” were constructed throughout the 20th century, driven by judicial and democratic inclinations towards including “abnormals” in public discourse. Thus, today’s observation of fat ladies aims to humanize rather than demonize them.

The photograph by Aguilar draws us into a familiar scene with her mimicry of the rock, yet a sense of displacement arises as we notice the grandeur of her naked body, an element conventionally unexpected in such a context. From this juncture, the question naturally arises: what emotions or modes of perception are truly evoked when we contemplate Aguilar’s portrayal? Are we inclined to humanize her, to label her as a monster, or perhaps something entirely different?

Jones (2012) suggests that while we may recognize ourselves in the artist’s photographs, akin to empathy, Aguilar appears to seek something more by evoking a sense of strangeness and embracing the disjointedness of her body. Thus, it’s possible to acknowledge the objectification of the body, as seen with performers, yet simultaneously, the artist draws attention to the marginalization of those with “striking” bodies. In this light, Aguilar emerges as more queer than freak.

As Russo (1995) suggests, the concept of the freak finds its roots in the cultural milieu of the 19th century, particularly within the intricately coded realm of entertainment. Here, bodies are not merely physical entities but objects to be observed, akin to pieces of art demanding specific modes of perception. However, unlike Laura Aguilar’s approach, which challenges established norms, the representation strategies employed in depicting fat ladies seem to paradoxically embrace the normalization of “monstrosity” through the adoption of conventional codes: attire, setting, mannerisms—all conforming to societal expectations. It’s as if these “monsters” are reluctantly permitted entry into this world, albeit relegated to its periphery. In contrast, Aguilar’s photography offers a different narrative: she seemingly reshapes the landscape, prompting us to contemplate the metaphorical denaturalization of established norms. Instead of conforming to a world that does not fully embrace her, the artist simply turns away. Eschewing makeup, elaborate hairstyles, and traditional feminine gestures, Aguilar presents herself in the nude. In terms of gender representation, Aguilar diverges from conventional ideals of femininity and attractiveness. Snider (2018) notes that Aguilar’s nudity exposes the unique inscription of her body—marked by factors such as fatness, disability, lesbianism, and Latina heritage. Aguilar embodies the unconventional, embracing the grotesque, and defying the constraints imposed by societal beauty standards.

It’s crucial to recall, as noted by Preciado (2010), that queer theory intertwines with the post-feminist movement, which includes notable figures like Judith Butler, Eve Kosofsky Sedgwick, Cherríe Moraga, and Donna Haraway. This intellectual current draws inspiration from North American interpretations of Lacan, Derrida, Lyotard, Deleuze, and Foucault. In this context, artists like Aguilar find themselves positioned to challenge essentialist notions more effectively, a stark departure from the constraints experienced by the freak show performers.

Despite the initial popularity of fat ladies, their presence gradually waned due to the emergence of medical and eugenic discourses, coupled with the industrialization of amusement parks. One of the final expressions of this phenomenon may have been Tod Browning’s film *Freaks* (1932), which featured characters with physical anomalies. However, the film was a commercial failure, with critics suggesting it should have been shown in a medical context rather than in cinemas (Courtine and Vigarello, 2011). By the 1960s, according to Bogdan (1988), the film found a new audience among camp enthusiasts. Concurrently, Goffman (1988) proposed the idea that abnormality was subjective, with stigma arising from the observer’s perspective, leading to the emergence of stereotypes. This period also saw a shift in perception, with deformity increasingly viewed as a social rather than purely physical phenomenon, affecting individuals’ right to social interaction and inclusion in the public sphere, thus acquiring psychological significance.

## 4 Results

The transition from the freak shows to the queer movements marks a significant shift in how marginalized bodies assert their presence. This transition is evident in the works of artists like Magalhães and Aguilar, who challenge normative standards and embrace the complexity of the fat body. Their artworks serve as a powerful critique of societal norms and emphasize the need for diverse representations in art and culture. This transition (from freak to queer) is notable as the bodies of these artists, once considered anomalous, extend beyond the confines of the stage, and permeate various spaces—from the streets to galleries, museums, and the digital realm. This shift allows us to perceive these artistic endeavors not solely as physical expressions but also as political statements, transcending conventional norms and embracing the antinormative in diverse spheres.

Amidst the myriad branches of the grotesque, the bodies of these artists, distorted and ambiguous, stand forth as embodiments of resistance. They are bodies that refuse obedience, defy classification, and disrupt the harmony of societal norms, rejecting the authoritarian dictates of conventional representations and challenging the various forms of fascism in all their guises. These fat, grotesque bodies of the artists refuse to be confined. In this sense, the results lead us to recognize in the artistic practices of Fernanda Magalhães and Laura Aguilar the essence of fat activism, which, according to Cooper (2007, 2008, 2010, 2013, 2016) and Snider (2010, 2013, 2017, 2018), involves rejecting the subjection of fatness to excessive medicalization and control, moral stratification, and commercial exploitation. Instead, it claims a political significance for the fat body, akin to the queer, expressing power and exposing the limitations of social constructions.

The political impact of the artists’ work does not just stem from their embrace of the fat body, considered grotesque in contemporary society. It also arises from the images and actions they create, which delve into the complexities of a phenomenon inherently transgressive. Consequently, the grotesque manifests in two ways: through corporeality and the reception of visual codes. These codes, entwined with a mixture of attraction and repulsion, blur boundaries, introduce incongruities, and uncertainties, prompting us to question the nature of deviation from norms.

Reflections on boundaries—of bodies, identities, genders, and nationalities—are crucial for understanding freaks, deemed aberrations for their deviation from the norm. These bodies, showcased on stages and elaborate sets, gained marvel status throughout the 19th century in Europe and the United States. However, perceptions of these bodies shifted at the turn of the century, coinciding with a strong condemnation of excessive fatness. Freak bodies closely resemble fat bodies; in fact, there was a specific category for them: fat ladies. While fat men also performed, often contrasting with the very thin, the ladies stood out as a phenomenon of immense popularity, often appearing alongside bearded women and dwarfs.

The political dimension of freak bodies, despite their role as entertainment, is noteworthy. Performers emphasized their differences, effectively “expanding the visual landscape,” as Garland-Thomson (2009) suggests, thereby highlighting inequalities, oppressions, and exploitations. While anatomical differences are typically stigmatized as ugly or undesirable, the artists’ appropriation of these insults serves as a potent political strategy, provoking shock and destabilization and thus capturing attention effectively.

Queer can be understood as an extension of the concept of freaks, making it the most suitable lens through which to interpret the artists’ works, emphasizing anti-normativity over neutrality. Their bodies and strategies transcend mere freakishness, embodying plurality, and the discourses of minorities. These manifestations extend beyond physicality, intersecting with various other differences, dissonances, and exclusions. In the case of Fernanda Magalhães’ *Rotundus*, the artist disrupts, embarrasses, and imposes her fatness on others. The absence of words for these unexpected actions is filled by the grotesque sensations of encountering something that, according to convention, should not exist: the monumental presence of the fat body.

The grandeur of the fat body is also a crucial factor in Laura Aguilar’s *Grounded #111*, serving as the main strategy in the image. Despite baring herself in public, Aguilar did not seek to captivate the audience; rather, she rebelled, showing disinterest, and turning her back. This approach, combined with her fusion with the earth and the majesty of her corpulence, nonetheless captures attention. In conclusion, it is important to emphasize the significance of visibility achieved through the grotesque. These artistic projects make marginalized bodies visible, creating tensions about what should or should not be shown or presented. The artists materialize corpulence and inscribe themselves into the public sphere, navigating the boundaries between the particular and the public, the beautiful and the ugly, the elevated and the degraded. They occupy, resist, and transgress with visual representations that embrace ambivalence, confusion, excess, and monstrosity. By deconstructing notions generated by dominant culture, they effectively disconcert the audience, refusing passivity. The various paths within the grand labyrinth of the grotesque converge on a singular point: the refusal to conform to the norm.

## 5 Discussion

### 5.1 Key findings of the study

This discussion explores the transition from freak shows to queer movements, focusing on the works of Fernanda Magalhães and Laura Aguilar. Their art challenges normative standards and embraces the complexity of the fat body, serving as powerful critiques of societal norms and emphasizing the need for diverse representations in art and culture.

### 5.2 Prior research and context

In the 19th and early 20th centuries, freak shows were popular entertainment that showcased bodies considered anomalous. These spectacles drew crowds fascinated by physical differences, but they also reinforced stigmas and marginalized those on display. These individuals were often objectified and dehumanized, seen merely as curiosities rather than as people.

The rise of queer theory in the late 20th century marked a significant shift in the perception and representation of marginalized bodies. Influenced by post-structuralist thinkers like Michel Foucault, Jacques Derrida, and Gilles Deleuze, queer theory challenged normative assumptions about gender and sexuality. Queer theory emphasizes the fluidity and performativity of identity, rejecting the notion that gender and sexuality are fixed or binary. This perspective provided a new way to view non-conforming bodies, celebrating diversity instead of pathologizing it.

In contemporary art, marginalized bodies challenge visual norms and highlight social inequalities. The grotesque, which blends attraction and repulsion, serves as a powerful tool in this resistance. Fernanda Magalhães and Laura Aguilar employ the grotesque to disrupt conventional standards of beauty and normalcy, compelling viewers to confront their own biases. By presenting bodies that defy easy categorization, these artists prompt society to question what is considered acceptable or desirable and to recognize the inherent value in all forms of bodily expression.

### 5.3 Novelty and contribution

The study highlights the transition from the historical spectacle of freak shows to contemporary queer art, demonstrating how marginalized bodies have reclaimed agency. Artists like Magalhães and Aguilar do not merely accept their categorization as “freaks” but actively challenge and redefine it through their work. Their art extends beyond physical spaces, infiltrating digital, street, and institutional realms, thus broadening the discourse on body politics and visibility.

This research integrates into the broader understanding of how marginalized bodies assert presence in public and artistic spheres. It advances current views by: (1) demonstrating the evolution from passive display to active resistance; (2) highlighting the role of the grotesque in challenging and redefining societal norms, and (3) emphasizing the intersectionality of queer art, which intersects with various other forms of identity and marginalization.

## 6 Conclusion

The transition from freak shows to queer movements marks a significant shift in how marginalized bodies assert their presence. The works of artists like Magalhães and Aguilar challenge normative standards and embrace the complexity of the fat body, serving as powerful critiques of societal norms. Their art underscores the importance of diverse representations and highlights the potential of the grotesque as a political tool. This discussion invites further exploration of these themes, encouraging a deeper understanding of the intersectionality and transformative power of queer art.

This study has demonstrated how grotesque aesthetics function as a mode of resistance in queer artistic practices, directly challenging hegemonic identity norms and the mechanisms of exclusion that regulate corporeal visibility. By examining the works of Fernanda Magalhães and Laura Aguilar through the lens of queer theory and the sociology of deviance, we have reinforced the argument that normativity operates both through disciplinary structures and cultural discourse, as theorized by Foucault (2001) and Goffman (1988). These findings confirm the premise that the grotesque serves as a form of transgression and as an active political tool that reclaims agency over marginalized bodies.

Furthermore, the research questions guiding this study—particularly those concerning the role of grotesque representations in contesting normative ideals—have been addressed through a critical analysis of visual materials and cultural narratives. The historical trajectory from freak shows to contemporary queer art underscores a fundamental shift: while the former commodified and pathologized bodily difference, the latter reclaims and (re)signifies it as an act of defiance and self-determination. This underscores the continued relevance of theories on performativity (Butler, 2003) and biopolitical control (Preciado, 2010) in understanding contemporary artistic interventions.

These conclusions invite further exploration into the intersections of queer aesthetics, visual culture, and body politics, emphasizing the need for more research on how contemporary artists continue to negotiate visibility and resistance within the framework of normativity.

## Data Availability

The original contributions presented in the study are included in the article/supplementary material, further inquiries can be directed to the corresponding author.

## Footnotes

^1^“Cuir” is frequently present in Magalhães’ discourse and serves as a Brazilian alternative to the English term “queer.” As researcher Altmayer (2016) explains, cuir “[…] implies a resistance to the indiscriminate use of foreign terms, while also seeking greater proximity to the South American reality” (Altmayer, 2016, p. 252, translated by the author).

^2^The term, initially imbued with negative connotations and utilized by North Americans to denote Mexican descendants residing in the United States, underwent a transformative appropriation as a political force during the 1960s and 1970s by movements advocating for the affirmation of Latino culture (Anaya, 1998). Following a series of contentious debates within the realm of identity politics, the term has evolved into a theoretical construct, assuming a pivotal role in feminist discourses concerning “Mexican American” women (García, 1997). As a means of addressing gender inclusivity, it is also employed by certain factions as “chicanxs.”

^3^Although Bakhtin’s (1987) analyses primarily focus on medieval and Renaissance popular culture, they offer valuable insights that resonate with the works of Aguilar and Magalhães. Particularly noteworthy is Bakhtin’s notion that the images produced in these contexts are inherently ambivalent and deformed, even monstrous and horrendous, when viewed through the lens of “classical” aesthetics—the aesthetics of pre-established and complete everyday life. This observation sheds light on the fluid connections between historical cultural phenomena and modern artistic expressions, challenging conventional notions of beauty and perfection.

^4^In this research, gender is understood as a socially constructed category (Butler, 2003). In their latest work, Butler (2024) contends that while the ambiguity of the term “gender” is often praised as an example of postmodern fluidity, this very ambiguity makes it susceptible to becoming an “empty signifier.” This vulnerability allows it to be co-opted by right-wing political discourse and religious dogmatism, particularly within a global landscape characterized by precarity and misinformation. Like gender, the terms “woman” and “feminine” are also socially constructed categories that have been naturalized through historical and cultural processes. This perspective was already anticipated by philosopher and feminist Simone de Beauvoir in *The Second Sex—Facts and Myths* (1980, first published in 1949), with her famous statement: “One is not born, but rather becomes, a woman.”

^5^It is worth to highlight that Butler proposes the term “co-construction” to move beyond the binary framework, as human lives, indeed any life, “are born into a state of dependency that makes their life processes social from the beginning” (Butler, 2024, p. 210).

^6^Available at: https://www.youtube.com/watch?v=0DDknRfJBZU. Last accessed on: May 12, 2024.

^7^Diane Arbus, Circus Fat Lady with Her Pet Dog, 1964. Gelatin silver print, 20.3 × 19 cm. Available at: https://www.artnet.com/artists/diane-arbus/circus-fat-lady-with-her-pet-dog-X7vdR6WJAy1PY0OKjrjpBQ2.

^8^According to researcher Santos (2006), Arbus’s photographs can be understood as “anti-portraits” due to the realism that imbues them with an aesthetic bordering on the grotesque.

^9^According to Magalhães (2008), the technique she labels as *contaminated photographs* entails manipulating images by adding elements, drawings, collages, and other modifications.

^10^In the piece, the artist blends Dadaist aesthetics with abstraction, creating a sense of disorientation through visual “noise.”

^11^According to art historian Athanassoglou-Kallmyer (2018), ugliness encompasses everything that defies categorization: the irrational, the mundane, deformity, disorder, the marginal. This concept closely parallels attempts to understand the grotesque.

^12^Hannah Höch, Aus der Sammlung: Aus einem ethnographischen Museum, 1929. Collage, 26.2 × 17.5 cm. Available at: https://www.nationalgalleries.org/art-and-artists/31343/aus-der-sammlung-aus-einem-ethnographischen-museum-collection-ethnographical-museum. Last accessed on: May 18, 2024.

^13^Laura Aguilar, *Grounded #111*, 2006. Photograph. 36.83 × 38.1 cm. Available at: https://www.kcet.org/shows/artbound/connected-to-the-land-the-work-of-laura-aguilar.
